# DFT Study of the Oxygen Reduction Reaction Activity on Fe−N_4_-Patched Carbon Nanotubes: The Influence of the Diameter and Length

**DOI:** 10.3390/ma10050549

**Published:** 2017-05-18

**Authors:** Xin Chen, Rui Hu, Fan Bai

**Affiliations:** The Center of New Energy Materials and Technology, College of Chemistry and Chemical Engineering, Southwest Petroleum University, Chengdu 610500, China; hurui720@163.com (R.H.); baifan19960404@163.com (F.B.)

**Keywords:** Fe−N_4_ catalytic site, carbon nanotubes, oxygen reduction reaction, DFT

## Abstract

The influences of diameter and length of the Fe−N_4_-patched carbon nanotubes (Fe−N_4_/CNTs) on oxygen reduction reaction (ORR) activity were investigated by density functional theory method using the BLYP/DZP basis set. The results indicate that the stability of the Fe−N_4_ catalytic site in Fe−N_4_/CNTs will be enhanced with a larger tube diameter, but reduced with shorter tube length. A tube with too small a diameter makes a Fe−N_4_ site unstable in acid medium since Fe−N and C−N bonds must be significantly bent at smaller diameters due to hoop strain. The adsorption energy of the ORR intermediates, especially of the OH group, becomes weaker with the increase of the tube diameter. The OH adsorption energy of Fe−N_4_/CNT with the largest tube diameter is close to that on Pt(111) surface, indicating that its catalytic property is similar to Pt. Electronic structure analysis shows that the OH adsorption energy is mainly controlled by the energy levels of Fe 3d orbital. The calculation results uncover that Fe−N_4_/CNTs with larger tube diameters and shorter lengths will exhibit better ORR activity and stability.

## 1. Introduction

In recent years, the development of nonprecious metal catalysts with high oxygen reduction reaction (ORR) activity and stability has been a major focus of fuel cell research. As early as 1964, Jasinski discovered that metal−N_4_ macrocyclic compounds are able to catalyze the ORR [1]. Nevertheless, these metal macrocycle-based electrodes (such as Fe-phthalocyanine and Fe-porphyrin) have not shown yet any acceptable ORR efficiency and stability in practical use [2]. The main reason is that previous methods employed simple mixing of metal-macrocycle with carbon black, which resulted in poor mechanical contacts with carbon support and thus inferior electrical conductivity [3]. Subsequent studies have shown that pyrolysis of transition metal, carbon, and some nitrogen containing materials—such as organic complexes, nitrogen containing salts and even gaseous nitrogen precursors—can also produce active ORR sites [4]. It was proposed that the active sites are the Fe ions coordinated by four nitrogen atoms, which are incorporated to graphitic carbon and have the same local structure of Fe-porphyrin catalysts [5]. These Fe−N_4_/C catalytic sites in graphitic carbon matrix were demonstrated to have excellent ORR catalytic performance [2,6,7,8,9]. Recently, graphene- and carbon nanotube (CNT)-based ORR catalysts containing transition metal and nitrogen attracted much research interest due to their excellent ORR activities [10,11,12,13]. The related research indicates that the activity of metal−N*_x_*/C catalytic sites in graphene matrix is mainly affected by metal types, the N-coordination number and matrix size [13]. However, for Fe−N*_x_* catalytic sites in CNT matrix, little is known about the relationship between the activity and the catalytic structures. The ORR activity and stability of CNTs are highly related to the tube diameter and length, which can obviously influence the geometrical and electronic structures of CNTs [14,15,16,17]. However, there is little related research reported so far. A systemic investigation of this issue will be helpful to build a reasonable Fe−N*_x_*/CNT model and to understand its catalytic activity towards ORR.

In this paper, we applied the density functional theory (DFT) method to study how the tube diameter and length influence the ORR activity and stability of Fe−N_4_/CNTs nano-catalysts. The theoretical calculations indicate that Fe−N_4_/CNT catalysts with large tube diameter and short length should have good ORR activity and stability.

## 2. Computational Methods and Models

The DFT calculations carried out in this study were based on GGA/BLYP [18] using the Amsterdam Density Functional (ADF) program package [19]. ADF calculations were run using the DZP basis set keeping the 1s core of C, N, and O, and 1s–3p core for iron atoms frozen. Note that we did not consider the d-band correction for Fe atom. For all stationary states, spin multiplicity was allowed to relax: possible geometries with varying spin states were carefully checked and the ground state was determined to be the one with the lowest electronic energy.

The models used in this work are armchair nanotubes terminated with C−H bonds, and they are denoted as Fe−N_4_ (*N*, *N*)-*L*. Here, (*N*, *N*) represents the diameter of the CNT and *L* indicates the length (*N* = 2–8, *L* = 9.8, 12.3, 14.7, 17.3, 19.7, and 22.1 Å, respectively). From Fe−N_4_ (2, 2)-*L* to Fe−N_4_ (8, 8)-*L*, the diameters are 2.8, 4.3, 5.6, 6.8, 8.2, 9.7, and 11 Å, respectively. From *L* = 9.8 to 22.1 Å, the length of the CNT was increased by adding a cell containing several six-membered rings (note that the entire findings in this work could only apply to the studied armchair nanotubes).

The adsorption energy of the ORR species is an important reference point for determining the activity and stability of an electrocatalyst. In this paper, the adsorption energy (AE) of the ORR species is calculated using the equation: AE(molecule) = *E*(catalyst−molecule system) − *E*(catalyst) − *E*(molecule). Therefore, a negative adsorption energy suggests that the ORR species would be energetically favorable to be adducted to the Fe−N_4_ (*N, N*)-*L* catalysts. 

## 3. Results and Discussion

### 3.1. The Stability of Fe−N_4_ Catalytic Sites in Acid Medium

**Figure 1:**
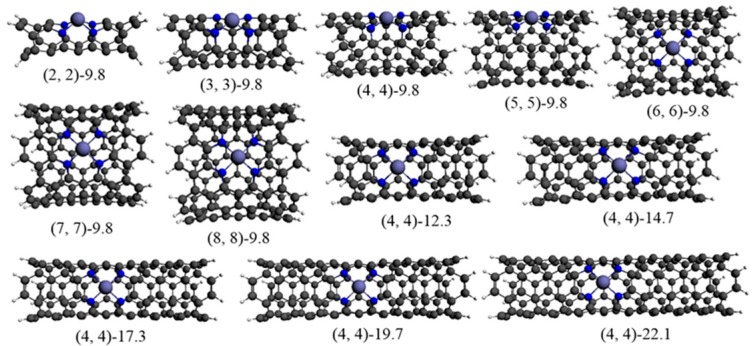
Optimized structures of Fe−N_4_ site in CNTs.

The optimized configurations of Fe−N_4_/CNTs with different tube diameters (the tube length is 9.8 Å) and lengths (taken Fe−N_4_ (4, 4)-*L* as an example) are shown in Figure 1. In order to evaluate the stability of these Fe−N_4_ catalytic sites in acid medium, we calculated the energies required (Δ*E*) to remove the Fe ion from the bulk Fe−N_4_/CNT structures using the following equation 

Fe−N_4_ (*N*, *N*)-*L* + 2[H_3_O^+^(H_2_O)_2_] → 2H−N_4_ (*N*, *N*)-*L* + [Fe(H_2_O)_6_]^2+^ (1)

Obviously, a larger Δ*E* denotes a more stable structure of the Fe−N_4_ site in the catalytic process (note that this is only a thermodynamic approximation and does not address the issue on kinetics of bond breaking which are also important for stability, as well as that we only consider the stability of the Fe−N_4_ site, not the corrosion process of bulk carbons).

**Figure 2:**
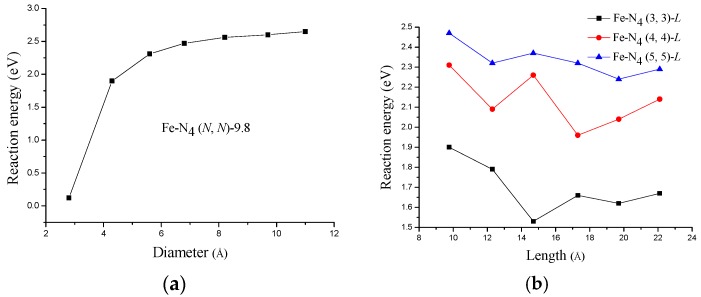
Reaction energies required for (**a**) Fe−N_4_ (*N*, *N*)-9.8 with different tube diameters (*N* = 2–8, the CNT length is 9.8 Å); and (**b**) Fe−N_4_ (*N*, *N*)-*L* with different tube lengths (*N* = 3–5, *L* = 9.8–22.1 Å).

The calculated Δ*E* values are shown in Figure 2. At a given length (such as 9.8 Å), as the tube diameter increases, the stability of Fe−N_4_ (*N*, *N*)-9.8 (*N* = 2–8) increases, as clearly shown in Figure 2a. However, as seen from the decreased Δ*E* values, the stability decreases with the increasing CNT length, as shown in Figure 2b. The Δ*E* is only +0.12 eV for Fe−N_4_ (2, 2)-9.8, indicating that it is not very stable in acid medium. This is mainly because the tube diameter is so small that the resulting hoop strain could significantly bend the Fe−N and C−N bonds. Therefore, the catalytic Fe site would be partially exposed to the external acid environment, leading to its instability. Except for Fe−N_4_ (2, 2)-9.8, other catalytic structures with tube length of 9.8 Å have higher Δ*E* values, suggesting they are stable in acid solution. Furthermore, for Fe−N_4_ (*N*, *N*)-*L* with a larger tube diameter (for example, the Fe−N_4_ (5, 5)-*L*), although the stability decreases with the increasing of tube length, the Δ*E* values are always above +2.2 eV, meaning that they are still stable in acid medium. It should be noted that the Δ*E* values in Figure 2b do not change monotonically with the increasing tube length. The possible reason for this is that the local geometrical structure (such as the average bond length of four Fe−N bonds) and surface electronic structure of Fe−N_4_/CNTs might be slightly affected by the tube length. Furthermore, although the curves are not completely monotonic, a general conclusion can also be made from Figure 2b that the Δ*E* values of longer structures are lower than those of shorter ones.

### 3.2. Adsorption of ORR Species

The adsorption energy (AE) of the ORR species is an important criterion in assessing the activity of a catalyst. Platinum-based materials are well known for their high catalytic activities for ORR. We can evaluate whether or not Fe−N_4_ (*N*, *N*)-*L* based catalysts have superior catalytic properties by comparing their adsorption energies with Pt.

**Figure 3:**
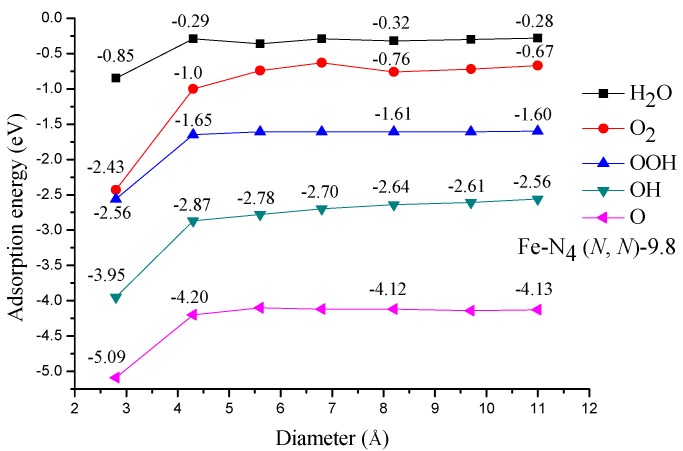
Calculated adsorption energies of the ORR species.

**Figure 4:**
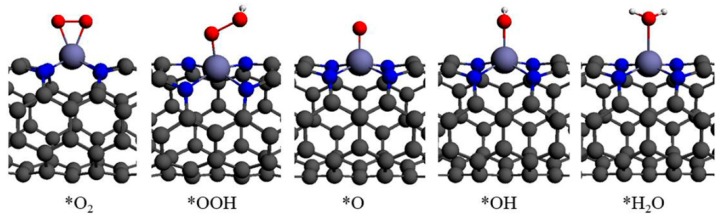
Calculated adsorption configurations of ORR species on Fe−N_4_ (4, 4)-9.8.

All the adsorption energies of the ORR species on Fe−N_4_ (*N*, *N*)-9.8 (*N* = 2–8) are shown in Figure 3, and only the adsorption configurations on Fe−N_4_ (4, 4)-9.8 are shown in Figure 4 for the sake of clarity. The AE(O_2_) on Fe−N_4_ (2, 2)-9.8 and Fe−N_4_ (3, 3)-9.8 are respectively −2.43 and −1.00 eV, while others are in the range of −0.63 to −0.76 eV. The experimentally determined low-coverage adsorption energy of O_2_ on Pt(111) is −0.3 to −0.5 eV [20,21,22], and theoretical values range between −0.41 and −1.04 eV depending on the methods and models used [23,24,25,26]. In general, the O_2_ adsorption energy on an ideal catalytic material should be as small as possible, but large enough to prevent O_2_ from drifting away or desorbing from the catalytic center [27]. The adsorption energy of O_2_ on Fe−N_4_ (2, 2)-9.8 is −2.43 eV, which is much stronger than that on the Pt(111) surface. As is well known, during the operation of the fuel cell, the energy loss of this non-electron-transfer step is unavailable for electrical work. The waste reaction energy at this step will lead to the reduction of the energy at those electron transfer steps. In this case, a large overpotential is inevitable. Furthermore, considering its instable nature in acid solution, a conclusion can be made that the Fe−N_4_ (2, 2)-9.8 is not a good catalyst for ORR. Conversely, other tubes with a length of 9.8 Å have suitable O_2_ adsorption energy, and are suitable for the ORR. 

The AE(OOH) on Fe−N_4_ (2, 2)-9.8, as shown in Figure 3, is −2.56 eV, which is very close to the value of O_2_ adsorption energy. Generally, for an effective electrocatalyst, the heats of formation of the OOH should be higher than the heats of formation of the adsorbed O_2_ on the surface, otherwise the ORR will not be an energetically favorable process [27]. Therefore, in view of this, the Fe−N_4_ (2, 2)-9.8 nanomaterial is again proven to be an ineffective electrocatalyst for ORR. Thus, the ORR catalyzed by Fe−N_4_ (2, 2)-9.8 is no longer considered in the following sections except when being specially pointed out. Interestingly, the AE(OOH) on other tubes almost has no change with the increasing tube diameter. The −1.60 eV adsorption energy seems to be unfavorable for ORR since it is slightly higher than the reported theoretical value of −1.06 to −1.16 eV for OOH adsorbed on Pt(111) surface [23,28]. However, as is well known, Pt is not a perfect catalyst for the ORR because it bonds OOH too weakly while it bonds O and OH too strongly [29,30]. Therefore, a higher OOH adsorption energy than Pt will lead to a larger heat loss during the first ORR step, which may facilitate the electron transfer. This conclusion is further confirmed by the calculated reaction energy for each ORR step, which we will show later. 

The adsorption energy of atomic O, similar to AE(OOH), does not change significantly with the increasing of tube diameter. However, the situation is quite different for OH adsorption. As is clearly shown in Figure 3, the AE(OH) gradually becomes weaker with the increasing tube diameter. Let us compare these OH adsorption results with those on Pt(111) surface. Previous DFT calculations of OH adsorption on Pt(111) surface led to AE(OH) = −2.26 to −2.45 eV [23,31]. DFT calculations performed on a 35 atom Pt cluster imitating the Pt(111) surface led to AE(OH) = −2.06 eV [32]. Therefore, the AE(OH) on the tubes with smaller diameter are relatively too strong, while on those tubes with larger diameters—for example, the Fe−N_4_ (8, 8)-9.8—are too close to those on Pt(111) surface. 

**Figure 5:**
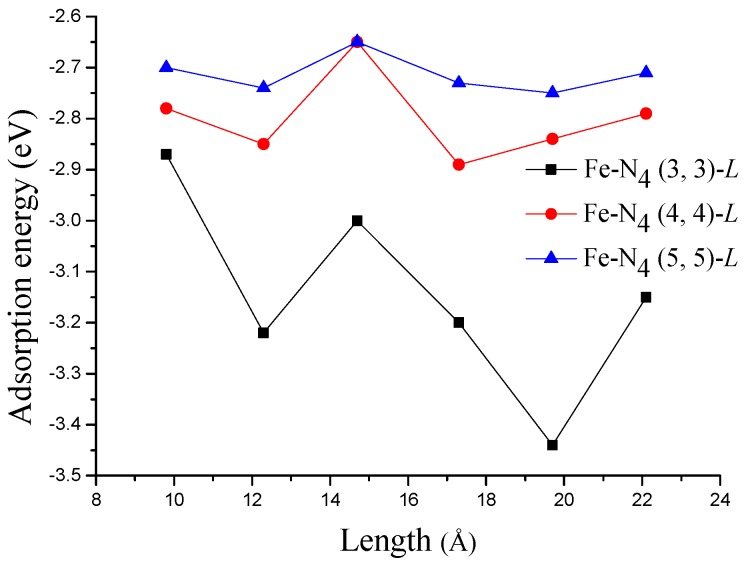
Calculated adsorption energies of OH with the increasing of tube length.

The variation of AE(OH) as a function of the tube length is shown in Figure 5. Unlike the case shown in Figure 3, it seems that there are no systematic changes with the increasing of tube length. The AE(OH) on Fe−N_4_ (4, 4)-*L* and Fe−N_4_ (5, 5)-*L* does not change much in the studied range of tube length, while on Fe−N_4_ (3, 3)-*L*, the change becomes obvious. The weakest AE(OH) on Fe−N_4_ (3, 3)-*L* is −2.87 eV, which is still much stronger than that on Pt(111) surface. The strong OH adsorption, as is well known, will increase the difficulty of removing OH from the catalyst’s surface and therefore lead to a high overpotential [33,34].

The calculated AE(H_2_O) on the Fe−N_4_ (*N*, *N*)-9.8 (*N* = 3–8) is in the range of −0.28 to −0.36 eV. These results are very close to the experimental values of −0.43 to −0.65 eV [35] and theoretical values of −0.22 to −0.60 eV on Pt(111) surface [23,36]. In general, the calculated adsorption energies of the ORR species on the above catalysts, especially of O and OH, are weaker than that on Fe−N_4_ embedded in the graphene [37] (note that this Fe−N_4_ was embedded in the bulk of graphene, not the edge). This may be attributed to the curvature effect of CNT [38]. Besides, we found that for all the studied Fe−N_4_ (*N*, *N*)-*L* (*N* = 3–8) nano-materials, the AE(H_2_O) are much weaker than their corresponding AE(O_2_). Generally, for an effective ORR catalyst, the H_2_O molecule should be adsorbed more weakly than O_2_, so that the catalytic cycle could repeat easily [39]. Thus, in principle, all the studied Fe−N_4_ (*N*, *N*)-*L* (*N* = 3–8) nano-materials have catalytic activities for ORR. Nevertheless, just as discussed above, only the tubes with larger diameters have AE(OH) close to the one on the Pt(111) surface. Since the OH group is regarded as a poison on the catalyst’s surface, stronger adsorption of OH on the tubes with smaller diameters will lead to higher overpotential for the ORR. Therefore, based on the above analysis, the Fe−N_4_ (*N*, *N*)-*L* based catalysts with large tube diameter and short length should have higher catalytic ORR activities.

In order to further support these conclusions, the relative energies for each electron transfer step are calculated, as shown in Table 1. The values on the Pt(111) are cited from the published literature [40]. The rate-determining step (RDS) is considered as the one with the smallest energy change. The higher the energy difference of the RDS is, the better the catalyst. Obviously, the RDS for all the structures is the reduction of OH group. From Fe−N_4_ (2, 2)-9.8 to Fe−N_4_ (4, 4)-9.8, the energy change values of RDS are positive, indicating these steps are energetically unfavorable. From Fe−N_4_ (5, 5)-9.8, due to the decreased OH adsorption, all the energy changes of the RDS become exothermic, suggesting the spontaneous nature of the process. As is expected, Fe−N_4_ (8, 8)-9.8 possesses the largest energy change of RDS, indicating it possesses the best ORR activity among all the screened catalysts. This conclusion is consistent with the analysis that the decreased OH adsorption is favorable for ORR, and the catalysts with larger tube diameters should have higher catalytic ORR activities.

### 3.3. Electronic Structural Effect on the ORR Activity

**Figure 6:**
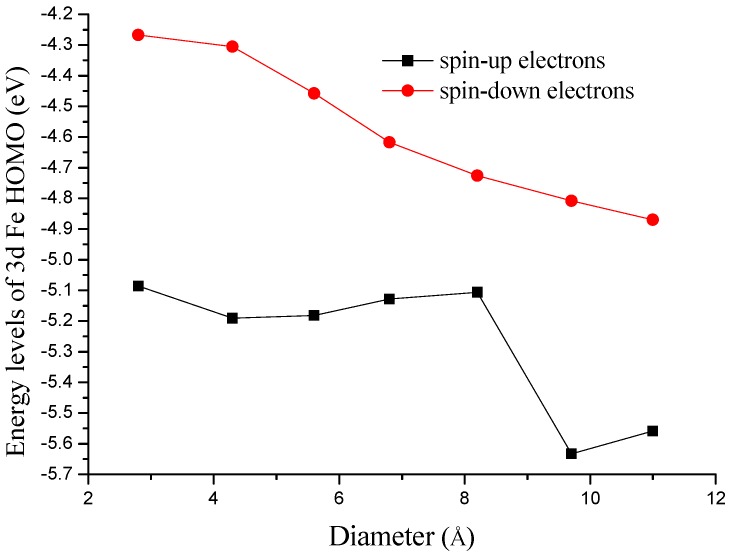
Calculated energy levels of Fe 3d HOMO with the increasing of tube diameter.

It is well known that the ORR activity is largely controlled by the electronic structure of the catalysts. DFT method is a good theoretical approach to investigate the relationship between the electronic structure and catalytic activity. Previous studies on periodic metal nanostructures, such as Pt-based materials [41,42] and other noble metals [43], show that the ORR activity is highly determined by the d-band center of the catalysts. For non-periodic molecular catalysts, such as various metal−N_x_/C materials, the occupied metal 3d orbital is proven to be of great significance in describing the catalytic activity [44]. In this paper, we calculated the energy levels of Fe 3d HOMO (the highest occupied molecular orbital) for both spin-up and spin-down electrons to investigate how orbital levels affect the ORR activity. The obtained results are shown in Figure 6. The energy levels of Fe 3d HOMO, especially for spin-up electrons, have been decreased with the increasing of tube diameter. Since the change of electronic structure is directly related to the variation of chemical reactivity, the adsorption energies of some intermediates will be affected. As shown in Figure 3, the AE(OH) is most affected by the electronic structure. Higher energy levels of Fe 3d HOMO could lead to stronger adsorption interactions. On the other hand, an appropriate HOMO orbital can induce an appropriate adsorption energy, which is favorable for ORR.

## 4. Conclusions

In this paper, the influences of diameter (2.8–11 Å) and length (9.8–22.1 Å) of carbon nanotube on Fe−N_4_/CNTs catalytic activities for ORR were investigated by theoretical calculations at the BLYP/DZP level of theory. The results indicate that the stability of the Fe−N_4_ site increases with the increase of tube diameter, but reduces with the increase of tube length. Besides, the tube with a too small diameter is not very stable in acid medium due to the fact that, at smaller diameters, the Fe−N and C−N bonds must be significantly bent due to hoop strain. The adsorption energy of the ORR species, especially of the OH group, becomes weaker with the increase of tube diameter. The tube with the largest diameter has the AE(OH) closest to that on the Pt(111) surface, suggesting a catalytic property similar to Pt. Electronic structure analysis shows that the AE(OH) is mainly controlled by the energy levels of Fe 3d orbital. It suggests that the Fe−N_4_ (*N*, *N*)-*L* nano-catalysts with large tube diameters and short lengths should have good ORR activity and stability. 

## Figures and Tables

**Table 1:** Calculated reaction energy changes (unit: eV) for each electron transfer step on Fe−N_4_ (*N*, *N*)-9.8 (*N* = 3–8) (* adsorbed species).

Reaction Step	(2, 2)	(3, 3)	(4, 4)	(5, 5)	(6, 6)	(7, 7)	(8, 8)	Pt(111)
O_2_ + H^+^ + e^−^ → * OOH	−2.50	−1.59	−1.55	−1.55	−1.55	−1.55	−1.54	−1.02
* OOH + H^+^ + e^−^ → * O + H_2_O	−1.89	−1.90	−1.85	−1.86	−1.87	−1.88	−1.88	−2.01
* O + H^+^ + e^−^ → * OH	−1.26	−1.07	−1.09	−0.99	−0.93	−0.88	−0.85	−0.77
* OH + H^+^ + e^−^ → H_2_O	+0.88	+0.21	+0.28	−0.37	−0.42	−0.46	−0.50	−0.88
